# New Gene Markers for Metabolic Processes and Homeostasis in Porcine Buccal Pouch Mucosa during Cells Long Term-Cultivation—A Primary Culture Approach

**DOI:** 10.3390/ijms19041027

**Published:** 2018-03-29

**Authors:** Marta Dyszkiewicz-Konwińska, Mariusz J. Nawrocki, Yan Huang, Artur Bryja, Piotr Celichowski, Maurycy Jankowski, Katarzyna Błochowiak, Katarzyna Mehr, Małgorzata Bruska, Michał Nowicki, Maciej Zabel, Bartosz Kempisty

**Affiliations:** 1 Department of Anatomy, Poznań University of Medical Science, 6 Święcickiego St., 60-781 Poznań, Poland; m.dyszkiewicz@ump.edu.pl (M.D.-K.); mjnawrocki@ump.edu.pl (M.J.N.); abryja@ump.edu.pl (A.B.); m.jankowski.14@aberdeen.ac.uk (M.J.); mbruska@ump.edu.pl (M.B.); 2 Department of Biomaterials and Experimental Dentistry, Poznań University of Medical Sciences, 61-701 Poznań, Poland; 3 OMFS IMPATH Research Group, Department of Imaging & Pathology, Faculty of Medicine, University of Leuven and Oral & Maxillofacial Surgery, University Hospitals Leuven, 3001 Leuven, Belgium; yanhuangcn@gmail.com; 4 State Key Laboratory of Oral Diseases, West China College of Stomatology, Sichuan University, 610041 Chengdu, China; 5 Department of Histology and Embryology, Poznań University of Medical Science, 6 Święcickiego St., 60-781 Poznań, Poland; pcelichowski@ump.edu.pl (P.C.); mnowicki@ump.edu.pl (M.N.); mazab@ump.edu.pl (M.Z.); 6 Department of Oral Surgery, Poznań University of Medical Sciences, 61-701 Poznań, Poland; kblochowiak@ump.edu.pl; 7 Department of Oral Rehabilitation, Poznań University of Medical Sciences, 61-701 Poznań, Poland; katarzynamehr@gmail.com; 8 Department of Histology and Embryology, Wrocław University of Medical Sciences, 50-367 Wrocław, Poland; 9 Department of Obstetrics and Gynecology, University Hospital and Masaryk University, 60177 Brno, Czech Republic

**Keywords:** oral mucosa, metabolic process, homeostasis

## Abstract

The oral mucosal tissue is a compound structure composed of morphologically and physiologically different cell types. The morphological modification involves genetically determined lifespan, which may be recognized as the balance between cell survival and apoptosis. Although the biochemical processes and pathways in oral mucosa, with special regards to drug transport, delivery, and metabolism, are well known, the cellular physiological homeostasis in this tissue requires further investigation. The porcine buccal pouch mucosal cells (BPMCs) collected from 20 pubertal crossbred Landrace gilts, were used in this study. Immediately after recovery, the oral mucosa was separated micro-surgically, and treated enzymatically. The dispersed cells were transferred into primary in vitro culture systems for a long-term cultivation of 30 days. After each step of in vitro culture (IVC), the cells were collected for isolation of total RNA at 24 h, 7, 15, and 30 days of IVC. While the expression was analyzed for days 7, 15, and 30, the 24th hour was used as a reference for outcome calibration. The gene expression profile was determined using Affymetrix microarray assays and necessary procedures. In results, we observed significant up-regulation of *SCARB1*, *PTGS2*, *DUSP5*, *ITGB3*, *PLK2*, *CCL2*, *TGFB1*, *CCL8*, *RFC4*, *LYN*, *ETS1*, *REL*, *LIF*, *SPP1*, and *FGER1G* genes, belonging to two ontological groups, namely “positive regulation of metabolic process”, and “regulation of homeostatic process” at 7 day of IVC as compared to down-regulation at days 15 and 30. These findings suggest that the metabolic processes and homeostatic regulations are much more intense in porcine mucosal cells at day 7 of IVC. Moreover, the increased expression of marker genes, for both of these ontological groups, may suggest the existence of not only “morphological lifespan” during tissue keratinization, but also “physiological checkpoint” dedicated to metabolic processes in oral mucosa. This knowledge may be useful for preclinical experiments with drugs delivery and metabolism in both animals and humans.

## 1. Introduction

The mammalian oral mucosal tissue is characterized by permanent morphological and biochemical modification during its lifespan. These changes are substantially regulated by the stage of the keratinization process that involves keratinoblasts. Moreover, the maintenance of the “balance” between keratinoblasts, keratinocytes, and fibroblasts is crucial for the morphological modification of oral mucosa [1]. The basic structure can be modified as the response to changes in environment, saliva contents, as well as drug delivery and administration. The proper morphology of oral mucosa influences the biochemical/metabolic status of the tissue. Therefore, the tissue architecture of oral mucosa and its cellular metabolic status are often recognized as the main factors determining the physiological and/or pathological condition of the oral mucosa [2]. We have recently intensively investigated the structure of oral tissue in pig using fluorescence observation and confocal microscopy. Using the primary cultivation systems we established the co-culture of mucosal keratinocytes and fibroblasts isolated from porcine buccal pouch mucosa. It is also well demonstrated that culture of separated keratinocytes may be achieved only using selective medium with enzymatic separation of tissue [3]. However, the culture system is often composed of both of these cells’ populations and therefore only co-culture system of oral mucosa may be successfully implemented in experiments.

Our recent studies, using microarray analysis, indicated substantial changes in gene expression during porcine buccal pouch mucosal cells culture [4]. We found that the transcriptomic profile was significantly related to the time period of in vitro culture (IVC). Moreover, using real-time cells proliferation system (RTCA) we found an increased proliferation index of mucosal cells cultured for long-term in vitro. These observation suggested that during long-term buccal pouch mucosal cells cultivation, the cells undergo substantial proliferation and differentiation [3]. The identification of new ontological groups that represent genes significantly up-, and/or down-regulated during cells proliferation in vitro suggested that oral mucosal cells represent tissue form recognized as “metabolic bioreactor”. Using microarray technique we analyzed both, known genes that are involved in new metabolic/homeostasis pathways, and new genes that may be markers of well recognized processes.

As we know, the cells’ morphological composition and metabolic/homeostasis status belong to the main features that describe tissue biology [5,6,7]. Although the morphological architecture of oral mucosa is well recognized using several histological methods, the metabolic versus biochemical status of these cells cultured primary in vitro, is not entirely known. Therefore this study was aimed to investigate the transcriptomic profile of genes involved in the metabolism and homeostasis in porcine buccal pouch mucosal cells during long-term primary in vitro culture. 

## 2. Results

**Figure 1:**
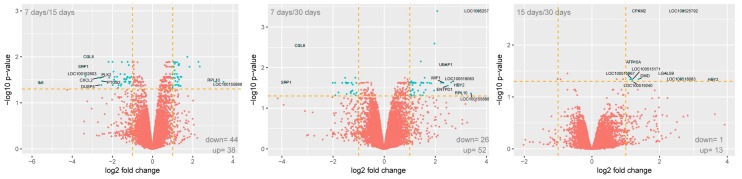
The Volcano plot representation between differently expressed genes between 7, 15, and 30 days of culture. The gene selection criteria were *p*-value beneath 0.05 and expression higher than two fold.

Using Affymetrix^®^ Porcine Gene 1.1 ST Array we examined expression of 12,258 porcine transcripts. Genes with fold change higher then abs (2) and wit corrected *p* value lower than 0.05 were considered as differentially expressed. This set of genes consists of 131 different transcripts. The amounts of up and downregulated genes were presented as volcano plots (Figure 1).

**Figure 2:**
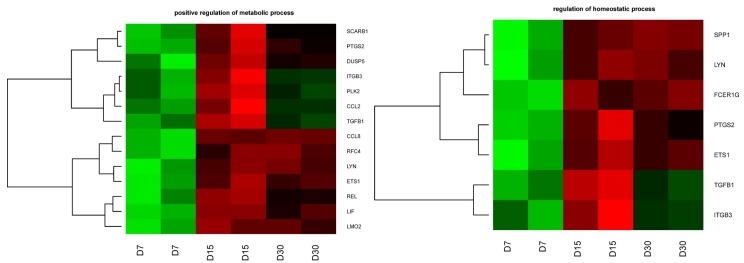
Heat map representation of differentially expressed genes belonging to the “positive regulation of metabolic process” and “regulation of homeostatic process”—GEOTERM BP database. Arbitrary signal intensity acquired from microarray analysis is represented by colors (green, higher; red, lower expression). Log2 signal intensity values for any single gene were resized to Row Z-Score scale (from −2, the lowest expression, to +2, the highest expression for a single gene). Each analysis was run in two samples coming from different in vitro cultures of oral mucosal cells. Changes in transcript levels were analyzed between three time periods-Day 7(D7), Day 15 (D15) and Day 30 (D30), of primary culture. The fold change was calculated in relation to transcript levels at hour 24 of primary culture.

DAVID (Database for Annotation, Visualization and Integrated Discovery) software was used for extraction of the genes belong to “positive regulation of metabolic process” and “regulation of homeostatic process” gene ontology biological process terms (GO BP). Up and down regulated gene sets were subjected to DAVID searching separately and only gene sets where adjusted *p*-value were lower than 0.05 were selected. Selected sets of genes were subjected to a hierarchical clusterization procedure and presented as heatmaps (Figure 2).

Set of the differentially expressed genes belonging to “positive regulation of metabolic process” and “regulation of homeostatic process” GO BP terms category were also presented with symbols, fold changes in expression, Entrez gene IDs and corrected *p* values were presented in Table 1.

**Figure 3:**
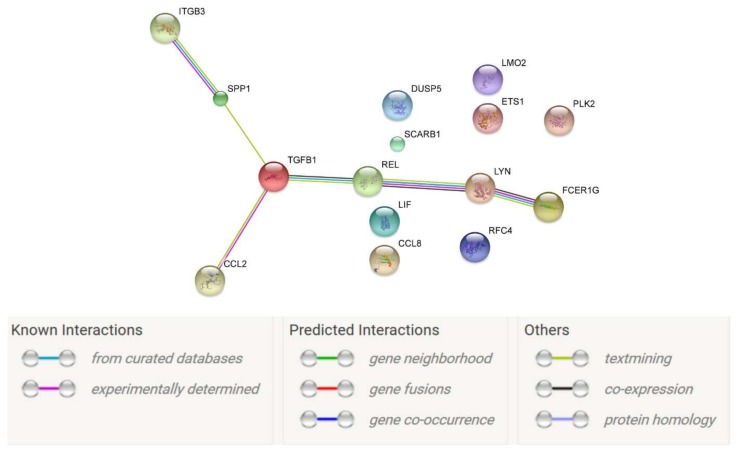
STRING (Search Tool for the Retrieval of Interacting Genes/Proteins)-generated interaction network among differentially expressed genes belonging to the “positive regulation of metabolic process” and “regulation of homeostatic process” GO BP terms. The intensity of the edges reflects the strength of interaction score.

STRING-generated interaction network was created with differentially expressed genes belonging to the “positive regulation of metabolic process” and “regulation of homeostatic process” ontology terms. The results show that there are evidences for *TGFB1* interaction with *REL, CCL2*, and *SPP1* genes. Moreover, evidence show that *REL* can interact with *LYN* and *FCER1G* genes and *SPP1* can interact with *ITGB3*. The results were shown in Figure 3.

Furthermore the *CPDB* analysis showed that two pairs of genes: *LYN* and *FCER1G* as well as *ITGB3* and *SPP1* can be found together in complexes categorized in Reactome, PID (Pathway Interaction Database) and BioCarta databases. The results were shown in Table 2.

**Figure 4:**
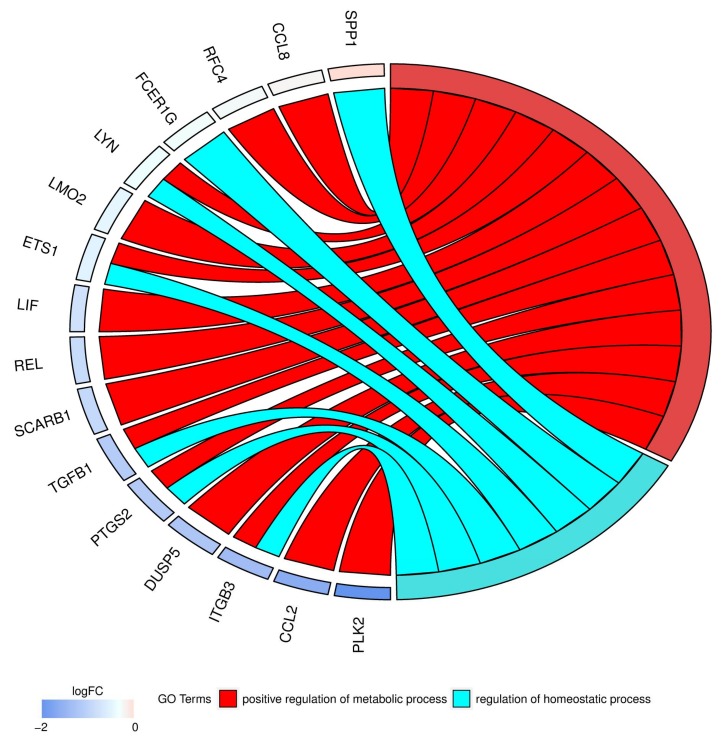
The representation of the relationship between differently expressed genes that belong to the “positive regulation of metabolic process” and “regulation of homeostatic process” GO terms. The ribbons show, which gene belongs to which categories. The genes were sorted by logFC from most to least changed gene. The gene that was downregulated the most, between day 7 and day 30, is presented on the bottom of the chart, the gene exhibiting the lowest amount of change is presented topmost. The intensity of the color indicates the scale of change in expression.

In Gene Ontology database genes that formed one particular GO group can also belong to other different GO term categories. By this reason we investigated genes shared between “positive regulation of metabolic process” and “regulation of homeostatic process” GO BP terms. The relations between these genes were showed in Figure 4.

**Figure 5:**
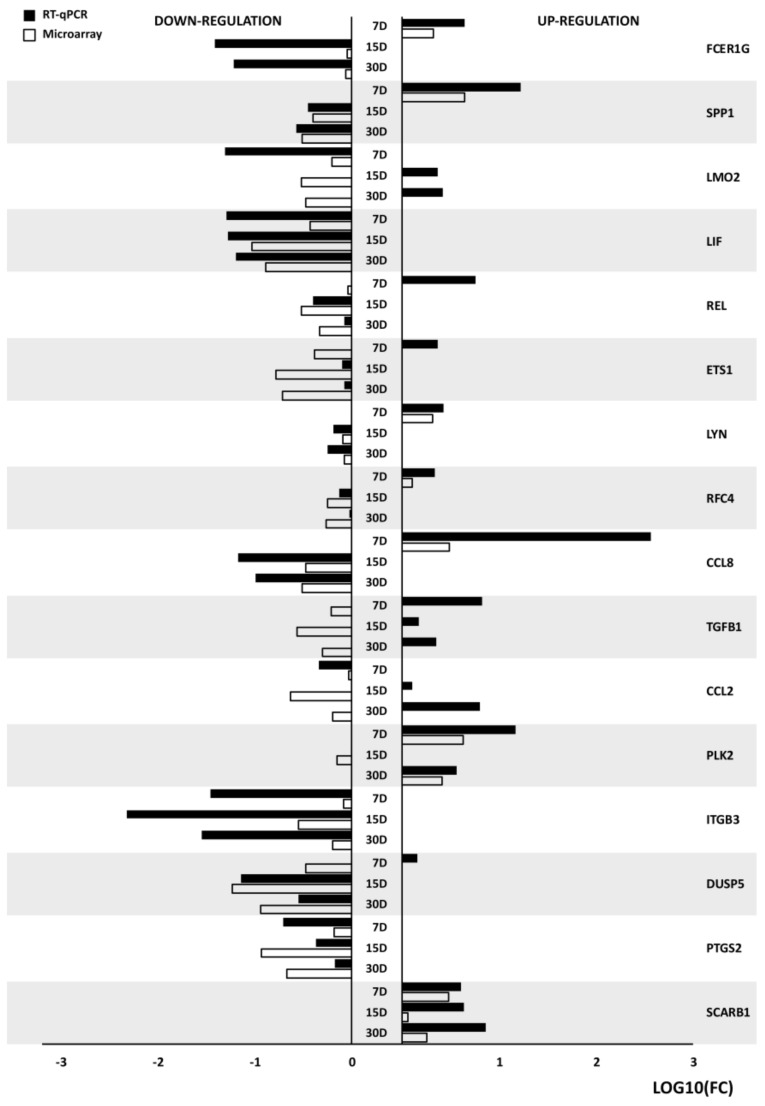
The results of RT-qPCR (Real Time-quantitative Polymerase Chain Reaction) validation of analyzed genes, presented in the form of bar-graph. All the fold changes were described in relation to the transcript levels in 24 h of primary culture. LogFC was used to present the data, in order to improve the clarity and comparability of up and downregulation results.

RT-qPCR (Real Time- quantitative Polymerase Chain Reaction) analysis was performed, in order to quantitatively validate the microarray analysis. The results were shown as a bar chart (Figure 5).

As can be seen in the figure above, while most of the directions of changes in gene expression have been validated, the scale of changes often varies between the methods. This is explainable as the RT-qPCR is a far more quantitative method than Microarrays. These variations are sometimes small (e.g., *SPP1*, *LIF*, *LYN*), while sometimes showing major discrepancies between the two analyses (e.g., *FCER1G*, *ITGB3*, *ETS1*). Nevertheless, in some examples the direction of changes also wasn’t validated by RT-qPCR. There were situations, in which only singular samples exhibited this kind of variations (e.g., *REL*, *ETS1*, *CCL2*), while in one example, *TGFB1*, all of the changes have shown different directions. This fact could be explained by difference in precision of those two methods, however it brings major limitation of whole transcriptome screening to light, as it is mainly a qualitative, rather than quantitative study.

## 3. Discussion

The intensity of morphological and biochemical changes of oral mucosa throughout its life is a characteristic feature of the analyzed tissue in mammals. The basic structure, with mutual proportions and interactions between keratinoblasts, keratinocytes, and fibroblasts, may be modified by many external factors, like environment changes, stress, mechanical strain, drug delivery, and administration. Along with the anatomical features, the oral mucosa plays a critical physiological role in distributing masticatory forces, as well as protecting the underlying residual ridge from excessive loading [8]. We can use mucosal biomechanical parameters, as fundamentals to define oral soft tissues behavior, which are closely relevant to clinical applications, including stimuli for tissue remodeling, pressure–pain thresholds, tissue displaceability and residual bone resorption. For example, the oral mucosa was found to be highly deformable and elastic under compression. Additionally, apart from the elastic response, there is a viscous component in this fluid-rich material that increases material viscoelasticity [9]. The processes associated with epithelium regeneration is the base for stem cell research and/or oral cancer investigation. These artificially cultured tissue equivalents are used in transplant surgery for the treatment of a variety of tissue dysfunctions in regions such as the eye, esophagus, or urethra [3,10]. Oral mucosa has been highlighted as a viable alternative source of epidermal cells, due to its easy preparation and suitable features, such as higher cell proliferation rates, lower terminal cell differentiation degrees and an increased biological activity as compared to epidermal keratinocytes. This tissue has another advantage. During harvesting it has been proven to produce less disability, and provides better aesthetic outcome [11,12]. As shown by Guzman-Uribe et al., it is possible to develop dermal-epidermal substitutes from the isolation of cells from oral mucosa for diabetic and healthy subjects using the air-liquid technique [13]. Nevertheless, the dynamic behavior of oral mucosa tissues remain not well understood.

Therefore, employing primary cell culture and a microarray approach, we aimed to investigate the transcriptomic profile of buccal pouch mucosal cells during long-term, in vitro culture. Our recent studies are an introduction to issues related to characterization of morphological and biochemical mechanisms of the oral mucosa [4,14]. In the present study, we examined the mRNA expression level changes during IVC of two ontology groups: (1) “Positive regulation of metabolic process” and (2) “Regulation of homeostatic process”. This approach will allow us to better understand dynamic behavior of oral mucosa tissues. From all analyzed genes, which showed different expression patterns, a total of 17 genes that belong to both gene ontology biological process terms were studied. In the group of described transcript profiles after microarray assay, we found five genes (*ITGB3*, *TGFB1*, *LYN*, *ETS1,* and *PTGS2*) with similar changes in transcripts expression profile during culture in both GO BP terms. Integrin subunit beta 3 (*ITGB3*) and transforming growth factor beta 1 (*TGFB1*) show increased transcript levels after 7 days of culture. During longer maintenance of the cell culture, after 15 days we observed significant decrease in *ITGB3* and *TGFB1* mRNA levels in both GO BP terms. Interestingly, in the final phase of our culture (after 30 days), heat map clearly shows slightly increased transcript expression. 

Integrins are heterodimeric cell surface glycoproteins consisting of α and β subunits, that connect the extracellular matrix (ECM) to the cytoskeleton [15]. Mechanical forces on matrix-integrin-cytoskeleton linkages are crucial for cell viability, morphology, and organ function. The production of force depends on the molecular connections from extracellular-matrix-integrin complexes to the cytoskeleton. Therefore, we can conclude that integrins are necessary to perform one of the key functions of the oral mucosa-distributing masticatory forces. Moreover, it is well recognized that integrins play an essential role in creating epithelial cell polarity [16,17,18]. Cell polarity is a fundamental organizing principle in metazoan that is necessary for cell division, differentiation, and morphogenesis. Polarization of epithelia is implicit in the development of lumens, which are essential for glandular tissues to carry out their normal functions, nevertheless loss of cell polarity and subsequent tissue disorganization is a hallmark of diseases such as cancer [19,20]. For example, β1 integrin ablation results in a loss of polarity leading to defective arterial lumen formation and asymmetric cell division in skin epithelia [21]. In other studies, authors using three-dimensional (3D) culture models demonstrated a direct role of β1-integrin in the regulation of epithelial cell polarity. Blocking β1-integrin function in this system caused inversion of apical polarity [22]. Other studies have shown integrins’ complex role in epithelial cell differentiation [23]. Some studies indicate pivotal role of integrins in the action of *TGFB1* signaling pathway in epithelial cells [24,25].

The cytokine transforming growth factor beta 1 (*TGFB1*) is a known mediator of fibroblast—myofibroblast differentiation, and it mainly elicits its effects through the SMAD (Mothers against decapentaplegic homolog) signal transduction pathway. It also influences a range of other cellular processes, including migration and proliferation, and its release initiates a sequence of events that are crucial in tissue repair, including chemoattraction of inflammatory cells, induction of angiogenesis, and regulation of inflammatory mediators [26]. The effects of *TGFβ* on transcription can be positive or negative depending on the targeted gene and the cellular context [27]. Cirillo et al. [28] suggested that the TGF-β family of cytokines secreted by cancer-associated fibroblast (CAFs) derived from genetically unstable oral squamous cell carcinomas promote the malignant phenotype by weakening intercellular epithelial adhesion. As shown by authors, members of the TGF-β family of cytokines distinguish CAFs from oral cancer and from normal oral fibroblasts [28]. In other studies, the researchers investigated the role of *TGF-β* in the induction of fibrosis in another oral disorder, namely oral submucous fibrosis (OSF) [29,30]. OSF is a potentially malignant condition of the oral cavity, characterized by a generalized submucosal fibrosis [31]. Fibrosis is caused by abnormal increase in the collagen production, nevertheless the exact mechanism is not known. Isoforms of *TGF-β*, *β1* and *β2* have been defined as a pro-fibrotic growth factors which cause deposition of extracellular matrix (ECM) by increasing the synthesis of matrix protein like collagen and decreasing the degradation by stimulating various inhibitor mechanisms. As detailed by Kamath et al., expression of *TGF-β1* and *TGF-β2* was increased in OSF as compared to normal oral mucosa [29]. Additionally, authors have shown the most prominent role of isoform β1 in the fibrotic pathway.

Similar patterns of mRNA expression exhibit also type of cytokine genes: C-C motif chemokine ligand 2 (*CCL2*) and polo like kinase 2 (*PLK2*)—protein kinases that have a role in normal cell division [32]. It should be noted that variable expression of *CCL2* and *PLK2* only concerns “positive regulation of metabolic process” GO term.

Proto-oncogenes *LYN* and *ETS1* have also shown similar changes in transcript expression profiles in both GO terms described. Heat maps indicates significant reduction of mRNA expression in day 15 compared with day 7. Subsequently, we demonstrated slightly increased transcript levels in 30 compare with D15, however still significantly lower than in D7 (see Table 1). Src-family protein-tyrosine kinases (STKs) belong to a family of nonreceptor-type tyrosine kinases and include at least eight members (including *LYN*) with significant amino acid sequence homology. STKs are known to play crucial roles in the regulation of leukocyte functions, including cell migration, adhesion, phagocytosis, and proliferation [33]. *LYN* is mainly expressed in hematopoietic cells with dual roles both as a positive and a negative signaling molecule in B lymphocytes and myeloid cells [34]. Lim et al. [35] also suggested that this proto-oncogene plays a positive regulatory role in retinoic acid-inducible gene I (*RIG-I*)—mediated interferon expression as a downstream component of IFN-β promoter stimulator-1 (*IPS-1*). As shown by Chen laboratory, *LYN* may be a key candidate gene for the formation of precancerous lesions in oral buccal mucosa [36].

The proto-oncogene *ETS1* is a transcription factor known to regulate the expression of a number of genes involved in extracellular matrix remodeling [37]. Similar to the proto-oncogene *LYN* described above, *ETS1* is a critical B cell transcription factor that prevents plasma cells (PCs) differentiation. Furthermore, as reviewed by Garrett-Sinha, *ETS1* is expressed in T cells, NK cells, and NK T cells and is inducible in other, non-lymphoid cell types in response to certain stimuli [37]. Interactions between *ETS1* and its important regulators *LYN*, which maintains *ETS1* expression to limit the differentiation of autoreactive PCs, were described by a Mayeux et al. [38] study. The researchers observed a significant genetic interaction between *Lyn* and *Ets1* in B cells, resulting in a greater and more rapid production of IgM autoAbs in *Lyn*+/−*Ets1*+/− mice than in *Lyn*+/− or *Ets1*+/− mice. Oikawa and Yamada indicate that generally, expression levels of ETS1 correlate well with the grade of invasiveness and metastasis. Upregulation of this ETS family member expression has been documented in many types of human tumors, including oral cancer [39]. Vairaktaris and coworkers compared the levels of *ETS1* expression in diabetic and normal oral mucosa rat models and subsequently they analyzed expression changes in cancerous stages. The authors have shown elevated expression of this proto-oncogene both in diabetic and normal rats, but in cancerous stages (oral squamous cell carcinoma (OSCC)) expression levels was higher in diabetic than in normal rats indicating that diabetes may contribute to enhanced invasion and metastatic potential by increasing *ETS1* levels [40]. In the hamster model with OSCC, the researchers also observed elevated expression levels of *ETS1* compare with controls [41].

Prostaglandin-endoperoxide synthase 2 (*PTGS2*), also known as cyclooxygenase 2 (*COX2*), is the last example of analyzed genes described in both GO BP terms. PTGS2 is the key, rate-limiting enzyme in prostaglandin biosynthesis from arachidonic acid, and acts both as a dioxygenase and as a peroxidase. Cyclooxygenases exist in at least two isoforms, PTGS1 (*COX1*) and PTGS2 (*COX2*). Unlike *PTGS1*, which is expressed constitutively, *PTGS2* expression is induced by cytokines and growth factors and is upregulated during inflammation [42]. *PTGS2* has been shown to be expressed in most solid tumor types [43,44]. Similar to the previously described genes, few studies analyzed *PTGS2* expression in OSCC. For example, Byatnal and coworkers evaluated *COX2* expression using indirect streptavidin biotin method. The researchers did not describe enzyme in normal oral mucosa. Elevated *COX2* expression was observed in 58 out of 75 specimens of OSCC [45]. Other studies have also shown, employing immune histochemical staining, that upregulated *COX2* expression was found in OSCC and dysplasia compared to normal mucosa subjects [46]. Additionally, Mauro et al. have compared both isoforms, *PTGS1* and *PTGS2*, expression levels by immunohistochemistry and RT-PCR in normal human oral mucosa and three different pathologies (hyperplasia, dysplasia, and carcinoma). As in the previously cited studies, *PTGS2* is not expressed in the normal tissue. Authors demonstrated enzyme expression in hyperplasia, reaches the maximum activation in dysplasia and then starts to be downregulated in carcinoma [47]. *COX1* mRNA and protein have been already detected in normal oral mucosa.

In conclusion, our data showed how morphological and biochemical changes of oral mucosal tissue throughout long-term cell culture in vitro are manifested in variable gene expression levels. However, it must be considered that this study is an entry level, in vitro analysis of the tissue of interest. Given the fact, that the cell culture is primarily obtained from the tissue sample, the results need to be accounted for all of the types of cells that are present in the “mix”, additionally the microarray approach, used to analyze the full transcriptome of the cells is largely qualitative, which can be seen, as validation of the results with quantitative RT-qPCR, often gives variable results. This might be due to the fact that the microarrays account for multiple available exons forming many variants of the expressed gene, which is not usually the case with RT-qPCR, as it probes for a specific gene sequence. It can also be explained with the mutual interaction between different cDNA species, present in the sample used for microarray analysis, which may lead to highly reproducible, false negative/positive results that impact the probe averages used for calculation of the total gene results. Finally, the analysis is focused only on transcriptome, which does not always reveal the full picture, as processes such as: alternative splicing, translation regulation, and post-translational modification can often change the amount in which the protein product is present, as well as the way in which it acts. Despite that and the fact that the study is in vitro based, which does not always translate into in vivo situation, it gives insight on the basic molecular mechanisms driving oral mucosal cells in vitro behavior, and may serve as reference for the future in vivo and clinical research. We observed differential expression profile of genes involved in two gene ontology groups, namely “positive regulation of metabolic process” (GO: 0009893) and “regulation of homeostatic process” (GO: 0032844). Firstly, we identified new genes, which may be the markers of these processes in oral mucosal cells in pigs. Additionally, differential expression profile of these cells during long-term in vitro culture suggests that the intensity of cellular metabolism and homeostasis is regulated by genes involved in “lifespan regulatory mechanisms”. Our recent experiments indicate that formation of proper morphological architecture of oral mucosa belongs to genetically inherited processes. Therefore, we suggested that, at least in vitro, both tissue cellular morphology and metabolic/physiological properties may be dependent on cellular lifespan. However, these results need to be confirmed by further analysis on protein level, possibly focused on particular, isolated cell populations found in the oral mucosal tissue.

## 4. Materials and Methods

### 4.1. Animals

For this study, a total of 35 pubertal crossbred Polish Landrace gilts (young female pigs), bred on commercial local farm were used. They had a mean age of 155 days (range 140–170 days) and the mean weight was 100 kg (95–120 kg). All of the animals were housed under identical conditions and fed the same forage (depending on age and reproductive status). All experiments were approved by Local Ethics Committee of the Poznan University of Life Sciences, Poland (permission no. 32/2012, 30.06.2012).

### 4.2. Cell Isolation and Culture

**Figure 6:**
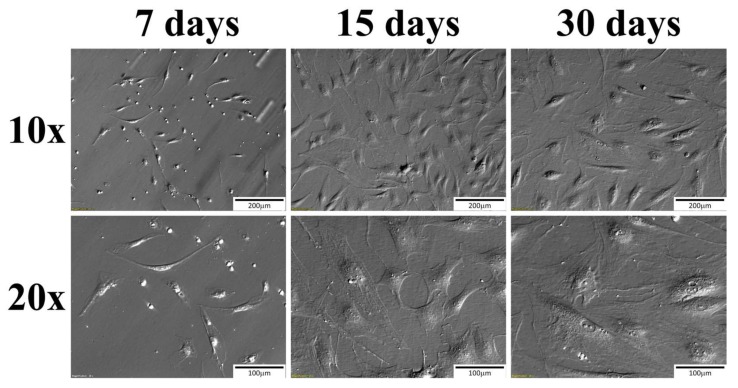
Photos of cell cultures prior to the material collection taken under an inverted microscope using relief contrast.

After slaughter, samples of buccal pouch mucosa were obtained within 40 min and transported to the laboratory. The excised tissue was washed twice in Dulbecco’s phosphate buffered saline (D-PBS) (137 mM NaCl, 27 mM KCl, 10 mM Na_2_HPO_4_, 2 mM KH_2_PO_4_, pH 7.4). The surface of the buccal pouch was surgically removed using sterile surgical blades. The tissue fragments were incubated with 0.05% collagenase I (Sigma Aldrich, Madison, WI, USA) for 40 min at 38 °C in a shaking water bath and then were treated witch 0.5% Trypsin/EDTA (Cascade Biologics, Portland, OR, USA) for 10 min. The cell suspension obtained from this digestion was filtered through mesh to remove non-dissociated tissue fragments. Isolated cells were washed three times by centrifugation (10 min at 200× *g*) with Dulbecco’s modified Eagle’s medium (DMEM; Sigma Aldrich, Madison, WI, USA) supplemented with gentamicin (20 μg/mL) and 0.1% BSA. The final cell pellet was resuspended in DMEM supplemented with 10% fetal calf serum (FCS; Sigma Aldrich, Madison, WI, USA) and 10 U/mL penicillin G, 10 mg/mL streptomycin, and 25 μg/mL amphotericin B. Cell viability was 90 to 95% as determined by trypan blue staining (Sigma Aldrich, Madison, WI, USA). The cells were cultured at 37 °C in a humidified atmosphere of 5% CO_2_. Once the oral mucosal cell cultures attained 70–80% confluency, they were passaged by washing with PBS, digested with 0.025% Trypsin/EDTA (Cascade Biologics, Portland, OR, USA), neutralized by a 0.0125% trypsin inhibitor (Cascade Biologics, Portland, OR, USA), centrifuged, and resuspended at a seeding density of 2 × 10^4^ cells/cm^2^. The culture medium was changed every three days. Before the collection of cells for the analyzed samples, photos of the culture were taken to monitor the possible changes of morphology (Figure 6). The reference sample photos (24 h) are not shown in the figure, due to the difficulties in obtaining clear photograph of a primary culture in such early stages, due to visual contaminants proprietary to such culture.

### 4.3. Microarray Expression Analysis and Statistics

Total RNA (100 ng) from each sample was subjected to two rounds of sense cDNA amplification (Ambion^®^ WT Expression Kit). The obtained cDNA was biotin labelled and fragmentated by Affymetrix GeneChip^®^ WT Terminal Labelling and Hybridization (Affymetrix, Santa Clara, CA, USA). Biotin-labelled fragments of cDNA (5.5 μg) were hybridized to the Affymetrix^®^ Porcine Gene 1.1 ST Array Strip (48 °C/20 h). Microarrays were then washed and stained according to the technical protocol using the Affymetrix GeneAtlas Fluidics Station. The array strips were scanned employing Imaging Station of the GeneAtlas System. Preliminary analysis of the scanned chips was performed using Affymetrix GeneAtlasTM Operating Software (Affymetrix, Santa Clara, CA, USA). The quality of gene expression data was confirmed according to the quality control criteria provided by the software. The obtained CEL files were imported into downstream data analysis software.

The primary microarray analysis was performed by the means of Bioconductor and R programming languages. The background was normalized by the Robust Multiarray Averaging (RMA) algorithm. Subsequently the microarray data was merged with a description file. To determine the statistical significance of the analyzed genes, moderated *t*-statistics from the empirical Bayes method were performed. The obtained p-value was corrected for multiple comparisons using Benjamini and Hochberg’s false discovery rate. The selection of significantly altered genes was based on a *p*-value beneath 0.05 and expression higher than two fold. 

Differentially expressed genes were subjected to selection by examination of genes involved in cell migration regulation. The differentially expressed gene list (separated for up- and down-regulated genes) was uploaded to DAVID software (Database for Annotation, Visualization and Integrated Discovery) [48]. Among extracted enriched Gene Ontology Biological Process (GO BP) terms, we focused on “positive regulation of metabolic process” (GO: 0009893) and “regulation of homeostatic process” (GO: 0032844).

Interactions between differentially expressed genes/proteins belonging to the “positive regulation of metabolic process” and “regulation of homeostatic process” GO terms were investigated by STRING10 software (Search Tool for the Retrieval of Interacting Genes) [49]. The list of gene names was used as a query for an interaction prediction. The search criteria were based on co-occurrences of genes/proteins in scientific texts (text mining), co-expression, and experimentally observed interactions. The results of such analysis generated a gene/protein interaction network where the intensity of the edges reflected the strength of the interaction score.

Additionally we have investigated if product (proteins) of selected differentially expressed genes belongs to a known protein complexes. Gene names were subjected to Max Plank Institute for Molecular Genetics Consensus Path Data Base (GCDB). This database integrates interaction networks in Homo sapiens including binary and complex protein-protein, genetic, metabolic, signaling, gene regulatory and drug-target interactions, as well as biochemical pathways from 32 public resources [50].

In order to further investigate the changes in studied GO terms, we have calculated the z-score (the number of up- regulated genes minus the number of down- regulated genes divided by the square root of the count) analysis with GOplot package [51]. The results shows allowed us to investigate the enrichment of those two GO BP terms.

### 4.4. Real Time q-PCR Analysis

The RT-qPCR method was performed to confirm the results obtained in the analysis of expression microarrays. Based on the results obtained during the analysis of expression microarrays, three genes were selected from each heatmap: the ones showing highest, lowest, and intermediate-level of expression. Changes in the level of expression of those genes were then examined (Table 1). Four biological samples of each gene were used for the analysis. Each biological test was performed in 3 replicates. Reverse transcription was based on the protocols and reagents of SABiosciences (RT^2^ First Stand Kit—330401), using a Veritimer 96 well Thermal Cycler. 1 microgram of each gene’s RNA transcript was used for reverse transcription. Real-time PCR was performed using the 7900HT Fast Real-Time PCR System (Applied Biosystems, Foster City, CA, USA), RT^2^ SYBR^®^ Green ROX^TM^ qPCR Master Mix (Qiagen Sciences, Hilden, Germany) and sequence-specific primers (Table 3).

Glyceraldehyde-3-phosphate dehydrogenase (*GADPH*), β-actin (*ACTB*), and hypoxanthine-guanine phosphoribosyltransferase *1* (*HRPT1*) were used as reference genes. Gene expressions were analyzed using the relative quantification (RQ) method. The q-PCR starters were designed using Primer3Plus software (http://primer3plus.com/cgi-bin/dev/primer3plus.cgi). The sequence of the respective genes was taken from the Ensemble database (http://www.ensembl.org/index.html), from which only the sequence of exons was exported, as the target sequence of the designed starter was spread across the border of two adjacent exons. This approach was used as a precaution against the possibility of a non-specific DNA template-based product (DNAse contained in the reverse transcription kit that was used as the other precaution). Agarose gel electrophoresis was applied to confirm the specificity of the amplified products.

## Figures and Tables

**Table 1:** The official gene symbols, fold changes, and adjusted *p*. values of the differently expressed genes that belong to the “positive regulation of metabolic process” and “regulation of homeostatic process”. Adjusted *p*-values are presented in brackets next to their respective fold changes.

Official Gene Symbol	Fold Change D7/D1	Fold Change D15/D1	Fold Change D30/D1	Fold Change D7/D15	Fold Change D7/D30	Fold Change D15/D30	ENTREZ GENE ID
*LMO2*	0.62 (0.004)	0.30 (0.0001)	0.33 (0.0002)	0.48 (0.03)	0.54 (0.03)	1.11 (0.74)	4005
*LIF*	0.37 (0.005)	0.09 (4.45 × 10^−5^)	0.13 (5.79 × 10^−5^)	0.25 (0.01)	0.35 (0.02)	1.40 (0.33)	3976
*REL*	0.91 (0.49)	0.30 (0.0004)	0.46 (0.002)	0.33 (0.02)	0.51 (0.06)	1.53 (0.38)	5966
*ETS1*	0.41 (0.002)	0.16 (0.0001)	0.19 (0.0002)	0.40 (0.03)	0.46 (0.05)	1.17 (0.75)	2113
*LYN*	2.04 (0.005)	0.81 (0.20)	0.83 (0.25)	0.40 (0.03)	0.41 (0.04)	1.03 (0.98)	4067
*RFC4*	1.27 (0.11)	0.57 (0.006)	0.54 (0.004)	0.45 (0.03)	0.43 (0.04)	0.95 (0.94)	5984
*CCL8*	3.04 (0.0005)	0.33 (0.0004)	1.30 (0.0003)	0.11 (0.01)	0.10 (0.01)	0.92 (0.86)	6355
*TGFB1*	0.61 (0.002)	0.27 (8.78 × 10^−5^)	0.50 (0.0005)	0.44 (0.01)	0.81 (0.22)	1.84 (0.07)	7040
*PLK2*	4.25 (0.002)	0.71 (0.20)	2.59 (0.01)	0.17 (0.03)	0.61 (0.31)	3.67 (0.19)	10,769
*ITGB3*	0.82 (0.32)	0.28 (0.001)	0.63 (0.04)	0.34 (0.04)	0.77 (0.42)	2.22 (0.23)	3690
*DUSP5*	0.34 (0.01)	0.06 (0.0004)	0.11 (0.0009)	0.17 (0.04)	0.34 (0.12)	1.97 (0.46)	1847
*PTGS2*	0.66 (0.16)	0.12 (0.0007)	0.22 (0.002)	0.18 (0.03)	0.33 (0.09)	1.86 (0.45)	5743
*SCARB1*	3.01 (0.001)	1.14 (0.40)	1.79 (0.01)	0.38 (0.03)	0.59 (0.13)	1.56 (0.36)	949
*FCER1G*	2.09 (0.001)	0.89 (0.32)	0.87 (0.24)	0.42 (0.03)	0.41 (0.02)	0.98 (0.98)	2207
*SPP1*	4.35 (0.002)	0.40 (0.01)	0.30 (0.005)	0.09 (0.02)	0.07 (0.02)	0.76 (0.77)	6696
*CCL2*	0.92 (0.75)	0.23 (0.002)	0.63 (0.09)	0.25 (0.04)	0.69 (0.40)	2.71 (0.23)	6347

**Table 2:** The list of proteins complexes from Reactome, PID (Pathway Interaction Database) and BioCarta databases, that included protein products of differently expressed genes that belongs to the “positive regulation of metabolic process” and “regulation of homeostatic process”.

*p*-Value	*q*-Value	Complex_Name	Source	Members_Input_Overlap	Members_Input_Overlap_Geneids	Size	Effective_Size
5.51 × 10^−6^	2.94 × 10^−5^	αv/β3 Integrin/Osteopontin	PID	ITGB3; SPP1	3690; 6696	3	3
1.10 × 10^−5^	2.94 × 10^−5^	GPVI:FceRI γ:FYN:LYN	Reactome	FCER1G; LYN	2207; 4067	4	4
1.10 × 10^−5^	2.94 × 10^−5^	αv/β3 Integrin/Osteopontin/Src	PID	ITGB3; SPP1	3690; 6696	4	4
1.83 × 10^−5^	2.94 × 10^−5^	Fc epsilon receptor I/LYN/SYK	BioCarta	FCER1G; LYN	2207; 4067	5	5
1.83 × 10^−5^	2.94 × 10^−5^	GPVI:phosphorylated Fc Epsilon R1 γ:FYN:LYN:Collagen type I:SYK	Reactome	FCER1G; LYN	2207; 4067	5	5
2.75 × 10^−5^	3.67 × 10^−5^	Antigen/IgE/Fc epsilon R1/LYN/SYK	PID	FCER1G; LYN	2207; 4067	6	6
3.85 × 10^−5^	4.40 × 10^−5^	Antigen/IgE/Fc epsilon R1/LYN/SYK/WIP	PID	FCER1G; LYN	2207; 4067	7	7

**Table 3:** Primer information and primer sequences used for the RT-qPCR analysis.

Gene	Number	Product Length (bp)	3′-5′	5′-3′
*SCARB1*	NM_213967.1	242	ccccatcgtctaccagatcc	agtcctgaagaagtggggtg
*PTGS2*	NM_214321	202	aaaggcctcaatcgaccaga	atctgggcgaggcttttcta
*DUSP5*	XM_003359366	250	tgcacgacccacctacacta	gcgagatcacactcctcctc
*ITGB3*	NM_214002.1	231	ctcatcggccttgctactct	agagacacccacaatcctgg
*PLK2*	XM_003133981	205	agcctgcttccagacaaaaa	gaaggaggtagagccgaggt
*CCL2*	NM_214214.1	112	gaagagtcaccagcagcaag	tggcttatggagtcctggac
*TGFB1*	NM_214015.2	208	accatgccaatttctgcctg	gaacgcacgatcatgttgga
*CCL8*	NM_001164515.1	206	caatggaaagatccccttca	ctcgcagtccaggtaggaag
*RFC4*	XM_013988325	169	atgcatctgatgaacgtgga	cgtcttaaagctgcctgagc
*LYN*	XM_021089363.1	248	agaggccatcaacttcggat	tctgcaggtagtcgaaggtg
*ETS1*	NM_001162886.1	166	atcagctggacaggagatgg	gtttacccgccgtcttgtg
*REL*	XM_005662527.3	238	ccagaaactgtggcaggatt	aggctgaggtaccattgtgg
*LIF*	NM_214402.2	209	gtgccaacgccctctttatt	attgaggctcctttggtccc
*SPP1*	NM_214023	82	agaagttccgcagatccgaa	tccgtctcctcactttccac
FCR1G	NM_001001265.1	111	accctcctctactgtcgact	ataagtctcctggttccggg
